# Evaluation of sexual function changes in female patients before and after cataract surgery

**DOI:** 10.1186/s12905-025-04061-y

**Published:** 2025-10-24

**Authors:** Şaban Kılıç, Emre Aydın, Çiğdem Deniz Genç

**Affiliations:** 1 Department of Ophthalmology, Samsun Education and Research Hospital, Kısla, Barıs Blv. No:199, İlkadım/Samsun, 55090 Turkey; 2 Department of Ophthalmology, Samsun University Faculty of Medicine, Samsun, Turkey

**Keywords:** Cataract surgery, Sexual function, FSFI, Visual health, Women, Quality of life

## Abstract

**Purpose:**

This study aimed to evaluate the changes in sexual function among women before and after cataract surgery, focusing on improvements in specific domains of sexual well-being.

**Methods:**

This study was conducted as a prospective observational study between August 1, 2024, and November 1, 2024, at the Samsun Training and Research Hospital. A total of 66 female participants aged 18 years or older, married, and actively engaging in sexual activities were included. Patients with pre-existing psychiatric or neurological disorders, previous diagnoses of sexual dysfunction, or those who refused to provide consent were excluded. The primary assessment tool was the Female Sexual Function Index (FSFI), which measures domains such as sexual desire, arousal, lubrication, orgasm, satisfaction, and pain. FSFI scores were collected both preoperatively and one month postoperatively. Other clinical parameters included age, BMI, educational level, and comorbidities such as hypertension and diabetes.

**Results:**

The mean age of the participants was 49.5 ± 4.4 years, and the average BMI was 24.0 ± 5.6 kg/m². Significant improvements were observed in all FSFI domains. Sexual desire increased from 2.3 ± 0.7 to 2.7 ± 0.8 (*p* < 0.001), and arousal improved from 1.9 ± 1.0 to 2.7 ± 1.3 (*p* < 0.001). Lubrication scores increased from 2.8 ± 1.2 to 3.2 ± 1.1 (*p* < 0.001), while orgasm scores improved from 1.7 ± 1.4 to 2.3 ± 1.4 (*p* < 0.001). Satisfaction scores rose from 2.5 ± 1.1 to 3.0 ± 1.1 (*p* < 0.001), and pain-discomfort scores improved from 1.9 ± 1.5 to 3.3 ± 1.6 (*p* < 0.001). The total FSFI score significantly increased from 13.0 ± 5.3 to 17.2 ± 5.1 (*p* < 0.001).

**Conclusions:**

Cataract surgery not only restores visual function but is also associated with improvements in female sexual function and well-being. Improvements in sexual desire, arousal, lubrication, and satisfaction highlight the broader benefits of improved visual health. These findings suggest that addressing visual impairments may contribute to improved sexual function and quality of life.

## Introduction

Cataracts are a common eye condition, particularly among older adults, characterized by the loss of transparency in the lens, which adversely affects vision [1]. Cataract surgery is one of the most frequently performed surgical procedures worldwide, and is used to restore vision. Regaining visual ability enhances the quality of life and promotes greater independence in performing daily activities. Good visual function not only boosts self-confidence but also positively influences social interactions and psychological well-being [2].

Women’s sexual function is influenced by biological, psychosocial, and environmental factors [3]. Visual function plays a crucial role in interpersonal relationships and in sexual health. Visual cues, such as facial expressions, gestures, and eye contact, are key triggers for sexual arousal and effective partner communication. Research has demonstrated that visual impairment is associated with higher rates of depression and anxiety, which are well-known psychosocial stressors that contribute to sexual dysfunction in women [4]. For instance, a comprehensive review found that cataract surgery frequently leads to significant mental health improvements among elderly patients, including reductions in depressive and anxiety symptoms [5]. Moreover, vision-related loss has been shown to undermines everyday functioning and quality of life, often resulting in emotional distress and compromised well-being [6]. Given these interconnections, restoring vision through cataract surgery may yield indirect yet meaningful improvements in female sexual function by alleviating psychological burdens and enhancing overall life satisfaction, strengthening the rationale for investigating this relationship. Despite these well-established connections between vision, psychological well-being, and sexual health, there is a paucity of studies specifically addressing how cataract surgery influences women’s sexual function.

This study aimed to evaluate the changes in sexual function in female patients before and after cataract surgery. By analyzing the impact of surgical intervention on women’s levels of sexual desire, arousal, and satisfaction, the relationship between eye health and sexual life was explored.

## Materials and methods

This study was designed as a prospective, single-center, observational study conducted at the Samsun Training and Research Hospital between August 1, 2024, and November 1, 2024. Female patients scheduled for elective cataract surgery were recruited consecutively. Data were collected at two time points: (1) preoperatively, to establish baseline sexual function, and (2) one month postoperatively, to evaluate the impact of surgery on sexual function. Female patients who underwent elective cataract surgery were included in this study. Data were collected in two stages: preoperatively, to assess patients’ baseline sexual function, and postoperatively, to evaluate the impact of surgery on sexual function. This study was approved by the Non-Interventional Clinical Research Ethics Committee of Samsun University (Session Date: 14/08/2024, Session Number: 2024/14). All participants provided written informed consent prior to their inclusion in this study. This study was conducted in accordance with the ethical principles outlined in the Declaration of Helsinki. Participant confidentiality was strictly maintained, and all personal data were anonymized before the analysis.

### Participants

The study population consisted of female patients aged ≥ 18 years, married, and actively engaged in sexual activities prior to surgery. Patients with psychiatric or neurological disorders or those who were previously diagnosed with sexual dysfunction were excluded from the study. Additional exclusion criteria included the lack of postoperative sexual activity and refusal to participate or sign the informed consent form.

## Data collection

The Female Sexual Function Index (FSFI) was the primary tool used to assess sexual function. The FSFI evaluates six domains of sexual function: desire, arousal, lubrication, orgasm, satisfaction, and pain. Each domain was measured preoperatively and postoperatively to observe any changes in sexual function due to the cataract surgery. Other demographic and clinical parameters collected included age, BMI, educational level (illiterate, primary/middle school graduate, high school graduate, university graduate or higher), and comorbidities such as thyroid disease, hypertension, and diabetes mellitus. The presence of hypertension (HT) and diabetes mellitus (DM) was also noted. The duration of surgery (in minutes) was recorded for all patients.

## Female sexual function index (FSFI) application

The Female Sexual Function Index (FSFI) was used as the primary instrument to evaluate sexual function among the participants. The FSFI is a validated, multidimensional self-report questionnaire consisting of 19 items that assess six domains of sexual function: desire, arousal, lubrication, orgasm, satisfaction, and pain [7]. Each domain is scored on a scale ranging from 0 to 1 to 5, with higher scores indicating better sexual functioning. The total FSFI score was calculated by summing the scores of each domain, yielding a range of 2–36. In this study, the FSFI was administered to the participants through face-to-face interviews both preoperatively and postoperatively. This approach ensured that the participants fully understood each question and provided accurate responses. The preoperative assessment established a baseline for sexual function, while the postoperative assessment, conducted after cataract surgery, evaluated any changes in sexual function attributable to the surgical intervention.

### Data analysis

The primary outcomes were changes in the FSFI total and domain-specific scores (sexual desire, arousal, lubrication, orgasm, satisfaction, and pain) between preoperative and postoperative evaluations. The data were analyzed to determine the impact of surgery on sexual function, with BMI and comorbidities as potential factors. Descriptive statistics were used to summarize patients’ demographic and clinical characteristics. Comparative analyses (e.g., paired t-tests) were conducted to assess the significance of the differences between the pre- and postoperative FSFI scores. All data were collected through face-to-face interviews to ensure accuracy and completeness, with attention given to maintaining the privacy and confidentiality of the participants.

### Statistical analysis

All statistical analyses were performed using SPSS version 27 (IBM Corp., Armonk, NY, USA). The normality of the data was evaluated using the Shapiro-Wilk test. Parametric or non-parametric tests were applied depending on the normality of the variables. For preoperative and postoperative comparisons of the same patients, the Paired Samples T-Test was used for variables with a normal distribution, while the Wilcoxon Signed-Rank Test was applied for non-normally distributed variables. In comparisons between two independent groups, the Independent Samples T-Test was used when the data followed a normal distribution, and the Mann-Whitney U Test was employed for non-normally distributed variables. When comparing more than two groups, One-Way ANOVA was used for normally distributed variables, whereas the Kruskal-Wallis Test was applied for non-normally distributed data. Descriptive statistics are presented as mean ± standard deviation (S.D.) for normally distributed variables and as median (interquartile range) for non-normally distributed variables. Categorical variables are reported as frequencies and percentages. Statistical significance was set at *p* < 0.05 for all analyses. In addition to the primary analyses, a post-hoc power analysis was conducted using G*Power version 3.1.9.7 (Heinrich-Heine-Universität Düsseldorf, Germany) to evaluate the study’s statistical power. For the paired t-tests (Lubrication and Pain-Discomfort domains), the achieved power was calculated based on the observed effect sizes. With 66 participants, the power was approximately 87% for lubrication (dz = 0.35) and greater than 99% for pain discomfort (dz = 0.90). These results indicate that the sample size was sufficient to detect differences.

## Results

The characteristics of the participants were analyzed, revealing a mean age of 49.5 ± 4.4 years. The average duration of the surgery was 19.7 ± 3.2 min, and the mean body mass index (BMI) was 24.0 ± 5.6 kg/m². In terms of educational level, 7.6% (*n* = 5) of the participants were illiterate, 40.9% (*n* = 27) had completed primary or secondary school, 40.9% (*n* = 27) were high school graduates, and 10.6% (*n* = 7) had a university degree or higher. Regarding comorbidities, 56.1% (*n* = 37) of the participants had no chronic diseases, whereas 43.9% (*n* = 29) had comorbid conditions, such as hypertension or diabetes (Table 1).

**Table 1:** Baseline characteristics of the study population

		Mean±S.D./Count (%)
Age (year)		49.5±4.4
Operation Time (min)		19.7±3.2
BMI (kg/m²)		24.0±5.6
Education Level	Illiterate	5 (7.6)
	Primary-Secondary School	27 (40.9)
	High School Graduate	27 (40.9)
	University and Above	7 (10.6)
Comorbidity	No	37 (56.1)
	Yes	29 (43.9)

Continuous variables are presented as mean ± standard deviation, and categorical variables are presented as number (percentage)

The results of the Female Sexual Function Index (FSFI) scores before and after cataract surgery are summarized in Table 2. Significant improvements were observed across all domains of sexual function after surgery. The mean sexual desire score increased from 2.3 ± 0.7 preoperatively to 2.7 ± 0.8 postoperatively (*p* < 0.001). Arousal scores improved from 1.9 ± 1.0 to 2.7 ± 1.3 (*p* < 0.001, Wilcoxon Signed-Rank Test), and lubrication scores increased from 2.8 ± 1.2 to 3.2 ± 1.1 (*p* < 0.001, Paired Samples T-Test). The orgasm domain also showed a significant increase from 1.7 ± 1.4 to 2.3 ± 1.4 (*p* < 0.001, Wilcoxon Signed-Rank Test). Satisfaction improved from 2.5 ± 1.1 to 3.0 ± 1.1 (*p* < 0.001, Wilcoxon Signed-Rank Test), and pain-discomfort scores increased from 1.9 ± 1.5 to 3.3 ± 1.6 (*p* < 0.001, Paired Samples T-Test). The total FSFI score significantly increased from 13.0 ± 5.3 preoperatively to 17.2 ± 5.1 postoperatively (*p* < 0.001, Wilcoxon Signed-Rank Test), indicating an overall improvement in sexual function following cataract surgery (Table 2).

**Table 2:** Changes in FSFI scores before and after cataract surgery

	Preop FSFI	Postop FSFI		
	Mean ± S.D.	Mean ± S.D.	*p* value	Cohen’s dz
Sexual Desire	2.3 ± 0.7	2.7 ± 0.8	< 0.001 ^b^	0.80
Arousal	1.9 ± 1.0	2.7 ± 1.3	< 0.001 ^b^	0.87
Lubrication	2.8 ± 1.2	3.2 ± 1.1	< 0.001 ^a^	0.63
Orgasm	1.7 ± 1.4	2.3 ± 1.4	< 0.001 ^b^	1.36
Satisfaction	2.5 ± 1.1	3.0 ± 1.1	< 0.001 ^b^	1.17
Pain-Discomfort	1.9 ± 1.5	3.3 ± 1.6	< 0.001 ^a^	1.02
Total Score	13.0 ± 5.3	17.2 ± 5.1	< 0.001 ^b^	1.42

Data are presented as the mean ± standard deviation. A: paired sample t test b: Wilcoxon Signed-Rank Test,

Table 3 highlights the key differences in FSFI scores across educational levels before and after cataract surgery. Postoperatively, participants with higher educational levels reported greater improvements in several domains. For example, sexual desire scores improved from 2.6 ± 1.0 preoperatively to 2.9 ± 0.9 among university graduates, though the difference between the groups was not statistically significant (*p* = 0.434). Arousal scores also increased significantly postoperatively, with the highest score reported by participants with university degrees (3.0 ± 1.0). Lubrication scores improved across all groups, with university graduates achieving the highest postoperative score (3.3 ± 0.8, *p* = 0.916). In terms of total FSFI scores, the preoperative mean ranged from 11.2 ± 1.4 among illiterate participants to 15.5 ± 8.3 among those with a university degree. After surgery, total scores improved across all groups, with university graduates achieving the highest mean score (19.3 ± 6.5, *p* = 0.463), reflecting an overall improvement in sexual function regardless of educational level (Table 3).

**Table 3:** FSFI scores according to educational level before and after cataract surgery

	Illiterate	Primary	High School	University Above	*p* value
Preop Sexual Desire	2.2 ± 0.3	2.1 ± 0.7	2.3 ± 0.7	2.6 ± 1.0	0.542^d^
Postop Sexual Desire	2.5 ± 0.8	2.5 ± 0.8	2.8 ± 0.9	2.9 ± 0.9	0.434 ^d^
Preop Arousal	1.4 ± 0.5	1.7 ± 1.0	2.1 ± 1.0	2.4 ± 1.3	0.151 ^d^
Postop Arousal	1.9 ± 0.8	2.6 ± 1.5	2.8 ± 1.1	3.0 ± 1.0	0.361 ^d^
Preop Lubrication	2.5 ± 0.8	2.6 ± 1.2	3.0 ± 1.2	2.9 ± 1.1	0.612 ^c^
Postop Lubrication	2.9 ± 0.9	3.2 ± 1.3	3.3 ± 1.1	3.3 ± 0.8	0.916 ^c^
Preop Orgasm	1.5 ± 1.0	1.5 ± 1.4	1.8 ± 1.3	2.4 ± 1.9	0.652 ^d^
Postop Orgasm	2.2 ± 0.9	2.2 ± 1.6	2.4 ± 1.2	3.0 ± 1.6	0.659 ^d^
Preop Satisfaction	1.9 ± 0.9	2.5 ± 0.9	2.6 ± 1.1	2.7 ± 1.7	0.623 ^d^
Postop Satisfaction	2.2 ± 1.1	3.0 ± 1.0	3.1 ± 1.2	3.3 ± 1.4	0.430 ^d^
Preop Pain-Discomfort	1.7 ± 0.5	1.8 ± 1.5	2.0 ± 1.5	2.5 ± 1.9	0.908 ^d^
Postop Pain-Discomfort	2.9 ± 1.1	3.4 ± 1.7	3.0 ± 1.6	3.9 ± 1.6	0.541 ^c^
Preop Total Score	11.2 ± 1.4	12.3 ± 4.6	13.9 ± 5.4	15.5 ± 8.3	0.674 ^d^
Postop Total Score	14.7 ± 2.4	17.0 ± 5.5	17.4 ± 4.7	19.3 ± 6.5	0.463 ^d^

Data are presented as the mean ± standard deviation. C: One-Way ANOVA, D: Kruskall-Wallis test

## Discusion

The findings of this study indicate that cataract surgery is associated with improvements in various domains of sexual function in female patients. Improvements were observed across all FSFI domains, including sexual desire, arousal, lubrication, orgasm, satisfaction, and pain relief. These enhancements indicate improvements across multiple FSFI domains, consistent with the notion that vision restoration may positively influence the quality of life. However, further studies are needed to clarify the potential effects on intimate relationships and psychological well-being. Although participants with higher educational levels showed numerically greater improvements in certain domains, such as arousal and total FSFI scores, these differences were not significant. Therefore, the observed variations should be interpreted with caution. Although the preoperative scores were relatively low, the notable postoperative improvements highlight the potential benefits of cataract surgery beyond vision restoration, highlighting the potential link between physical health and sexual function while acknowledging that causality cannot be established within the scope of this study.

Notably, the mean age of our study population was considerably younger than that reported in Western cohorts, such as those in the UK (76 years). According to World Health Organization data, the prevalence of diabetes among adults is 17% in Turkey, compared to 9% in the United Kingdom [8]. Similarly, the prevalence of obesity was higher in Turkey (33%) than in the UK (27%). These elevated metabolic risk factors may contribute to the earlier age at which patients in our population require cataract surgery.

Both our study and that of Cankurtaran et al. highlight the positive impact of cataract surgery on sexual function [9]. While Cankurtaran et al. found improvements in sexual desire and satisfaction primarily in patients with severe and profound visual impairment, our study demonstrated significant enhancements across all FSFI domains, including desire, satisfaction, and pain relief, regardless of the patients’ education level or comorbidities. Both studies support the conclusion that restoring vision improves overall sexual well-being and quality of life, indicating that cataract surgery provides benefits beyond visual recovery alone.

Organic conditions such as diabetes mellitus, cardiovascular diseases, penile disorders, and psychological issues such as depression and anxiety can negatively affect erectile function [10]. Similarly, disruptions in the five primary senses can impair both erectile function and sexual arousal [11, 12]. However, the impact of sensory impairments, particularly visual deficits, is often underestimated until the condition reaches an advanced stage. Visual stimuli play a vital role in triggering sexual arousal, and exposure to such stimuli enhances sexual desire [13]. Previous studies have demonstrated that visual function significantly influences sexuality and sexual satisfaction in both men and women, with comparable effects across genders [14]. Heine et al. and Strawbridge et al. noted that individuals with visual impairments may struggle to interpret visual cues such as eye contact, facial expressions, and gestures. The inability to perceive these nonverbal signals can impair functionality and diminish overall well-being [15, 16].

The study by Friberg et al. and our research both explored the relationship between vision and sexual health, albeit from opposite perspectives. In a study by Friberg et al., patients experienced a sudden loss of vision immediately following intense sexual activity, with no underlying predisposing factors identified [17]. This temporary visual impairment, caused by intraretinal, preretinal, or vitreous hemorrhages, disrupts visual function, although most patients recover spontaneously without lasting damage. The findings illustrate how sudden visual loss can be linked to sexual activity, underscoring the interplay between physical exertion and vision in this context. In contrast, our study focused on how restoring visual function through cataract surgery positively impacts women’s sexual health. Rather than impairing sexual well-being, as seen in Friberg et al.’s study, our findings highlight significant improvements in sexual function following cataract surgery, with enhancements in sexual desire, arousal, lubrication, satisfaction, and pain relief. This difference emphasizes the broader impact of visual health on intimate relationships: while sudden visual loss may hinder sexual activity and well-being, restoring vision through cataract surgery promotes sexual health and improves the overall quality of life. Both studies demonstrated the complex bidirectional relationship between vision and sexual function, showing that vision not only affects daily functioning but also plays a critical role in maintaining sexual well-being.

This study had several limitations that should be acknowledged. First, the prospective observational design of our study did not allow for the establishment of definitive causal relationships between cataract surgery and improvements in sexual function. Second, the sample size was relatively small and drawn from a single center, which may affect the generalizability of the findings to broader populations. Third, the study exclusively included married women who were actively engaged in sexual activity prior to surgery, potentially introducing selection bias and limiting the applicability of the results to unmarried women and those who were not sexually active. Fourth, patients with psychiatric or neurological disorders or those previously diagnosed with sexual dysfunction were excluded, which may further limit the generalizability of our findings. Fifth, the use of self-reported measures, such as the FSFI, may introduce reporting bias, as participants might underreport or overreport their sexual function due to personal or cultural factors. Finally, the relatively short duration between preoperative and postoperative assessments may not capture long-term changes in sexual function. Future studies with larger, more diverse populations and longer follow-up periods are needed to validate these findings and explore the underlying mechanisms linking cataract surgery to changes in sexual function. A limitation of our study is the short follow-up period; a longer-term evaluation is needed to confirm the persistence of improvements in sexual function.

## Conclusion

This prospective observational study demonstrated that cataract surgery not only restored visual function but also significantly improved female sexual function across all FSFI domains, including desire, arousal, lubrication, orgasm, satisfaction, and pain. These findings highlight the broader impact of vision restoration on quality of life and intimate well-being. The results suggest that addressing visual impairment may contribute to improved sexual health and psychosocial outcomes in women. Future studies with larger populations and longer follow-up periods are warranted to validate these results and explore the underlying mechanisms.

## Data Availability

The data that support the findings of this study are available from the corresponding author upon reasonable request.

## Abbreviations

ANOVA: Analysis of variance
BMI: Body mass index
DM: Diabetes mellitus
FSFI: Female sexual function index
HT: Hypertension
SD: Standard deviation
SPSS: Statistical package for the social sciences

## References

[1] Fukuoka H, Afshari NA. The impact of age-related cataract on measures of frailty in an aging global population. Curr Opin Ophthalmol. 2017;28(1):93–7. 27820747 10.1097/ICU.0000000000000338

[2] Signes-Soler I, Javaloy J, Montés-Micó R, Muñoz G, Montalbán R, Hernández A, et al. Vision-Related quality of life after cataract surgery in West Africa. West Afr J Med. 2023;40(3):329–35. 37018220

[3] Towe M, Huynh LM, El-Khatib F, Gonzalez J, Jenkins LC, Yafi FA. A review of male and female sexual function following colorectal surgery. Sex Med Rev. 2019;7(3):422–9. 31147295 10.1016/j.sxmr.2019.04.001

[4] Aslan E, Yılmaz B, Acar Z. Reproductive health, sexual function and satisfaction levels in women with physical, hearing, and visual disabilities. Sex Disabil. 2021;39(3):595–608.

[5] Dhawale KK, Tidake P. Cataract surgery and mental health: a comprehensive review on outcomes in the elderly. Cureus. 2024;16(7):e65469. 39188457 10.7759/cureus.65469 PMC11346754

[6] Chen PW, Liu PP, Lin SM, Wang JH, Huang HK, Loh CH. Cataract and the increased risk of depression in general population: a 16-year nationwide population-based longitudinal study. Sci Rep. 2020;10(1):13421. 32770101 10.1038/s41598-020-70285-7 PMC7414888

[7] Rosen R, Brown C, Heiman J, Leiblum S, Meston C, Shabsigh R, et al. The female sexual function index (FSFI): a multidimensional self-report instrument for the assessment of female sexual function. J Sex Marital Ther. 2000;26(2):191–208. 10782451 10.1080/009262300278597

[8] Noncommunicable Diseases Data Portal. Country Profile. World Health Organization. 2023 [cited 3 September 2025]. Available from: https://ncdportal.org/CountryProfile/GHE110/TUR

[9] Cankurtaran V, Ozates S, Ezerbolat Ozates M, Ozler S. Influence of visual acuity level on sexual function in patients with cataract. Indian J Ophthalmol. 2020;68(8):1579–83. 32709781 10.4103/ijo.IJO_2290_19 PMC7640845

[10] Heiman JR. Sexual dysfunction: overview of prevalence, etiological factors, and treatments. J Sex Res. 2002;39(1):73–8. 12476261 10.1080/00224490209552124

[11] Bakır S, Penbegül N, Gün R, Yorgancilar E, Kiniş V, Özbay M, et al. Relationship between hearing loss and sexual dysfunction. J Laryngol Otol. 2013;127(2):142–7. 23253624 10.1017/S0022215112002952

[12] Hatfield RW. Touch and human sexuality. Human sexuality: An encyclopedia. 1994:178 – 92.

[13] McCall K, Meston C. Cues resulting in desire for sexual activity in women. J Sex Med. 2006;3(5):838–52. 16942529 10.1111/j.1743-6109.2006.00301.x PMC2861288

[14] Salehi M, Azarbayejani A, Shafiei K, Ziaei T, Shayegh B. Self-esteem, general and sexual self-concepts in blind people. J Res Med Sci. 2015;20(10):930–6. 26929756 10.4103/1735-1995.172764 PMC4746865

[15] Heine C, Browning CJ. Communication and psychosocial consequences of sensory loss in older adults: overview and rehabilitation directions. Disabil Rehabil. 2002;24(15):763–73. 12437862 10.1080/09638280210129162

[16] Strawbridge WJ, Wallhagen MI, Shema SJ. Impact of spouse vision impairment on partner health and well-being: a longitudinal analysis of couples. J Gerontol B Psychol Sci Soc Sci. 2007;62(5):S315–22. 17906175 10.1093/geronb/62.5.s315

[17] Friberg TR, Braunstein RA, Bressler NM. Sudden visual loss associated with sexual activity. Arch Ophthalmol. 1995;113(6):738–42. 7786214 10.1001/archopht.1995.01100060064033

